# Acetonitrile­bis­(2-methyl-1,10-phenanthroline)copper(II) tetra­fluoridoborate

**DOI:** 10.1107/S1600536810038845

**Published:** 2010-10-02

**Authors:** Stephen P. Watton

**Affiliations:** a Department of Chemistry and Biochemistry, Central Connecticut State University, 1615 Stanley Street, New Britain, CT 06050, USA

## Abstract

In the title compound, [Cu(CH_3_CN)(C_13_H_10_N_2_)_2_](BF_4_)_2_, the fivefold-coordinate Cu^II^ atom is located on a twofold rotation axis, imposing twofold symmetry to the complete cation. The structure exhibits disorder of the anion, which was successfully refined using a two-site model with 0.810 (3):0.190 (3) occupancy. The methyl group of the acetonitrile ligand is likewise disordered, here about the twofold rotation axis in a 1:1 ratio.

## Related literature

For related structures, see: Watton (2009 ▶).
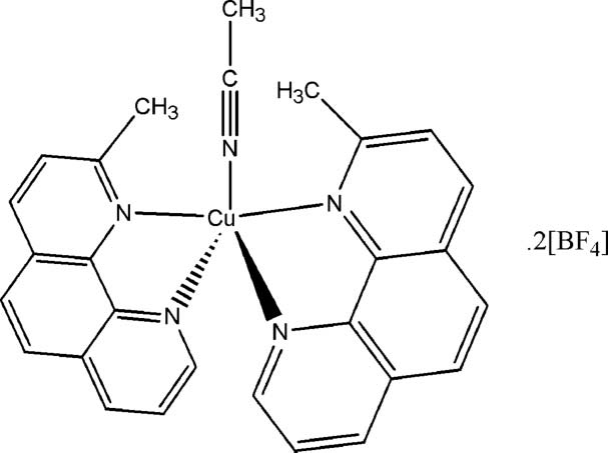


## Experimental

### 

#### Crystal data

[Cu(C_2_H_3_N)(C_13_H_10_N_2_)_2_](BF_4_)_2_
*M* *_r_* = 666.67
Monoclinic, 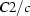

*a* = 25.0665 (11) Å
*b* = 8.8120 (1) Å
*c* = 16.8419 (14) Å
β = 131.824 (8)°
*V* = 2772.2 (3) Å^3^
*Z* = 4
Mo *K*α radiation
μ = 0.87 mm^−1^
*T* = 293 K
0.25 × 0.20 × 0.15 mm

#### Data collection

Oxford Diffraction Sapphire 3 CCD diffractometer
Absorption correction: multi-scan (*CrysAlis RED*; Oxford Diffraction, 2006 ▶) *T*_min_ = 0.784, *T*_max_ = 1
30296 measured reflections
5578 independent reflections
4442 reflections with *I* > 2σ(*I*)
*R*_int_ = 0.016

#### Refinement

*R*[*F* ^2^ > 2σ(*F* ^2^)] = 0.040
*wR*(*F* ^2^) = 0.120
*S* = 1.08
5578 reflections
219 parameters
6 restraints
H-atom parameters constrained
Δρ_max_ = 1.07 e Å^−3^
Δρ_min_ = −0.81 e Å^−3^

### 

Data collection: *CrysAlis CCD* (Oxford Diffraction, 2006 ▶); cell refinement: *CrysAlis RED* (Oxford Diffraction, 2006 ▶); data reduction: *CrysAlis RED*; program(s) used to solve structure: *SHELXS97* (Sheldrick, 2008 ▶); program(s) used to refine structure: *SHELXL97* (Sheldrick, 2008 ▶); molecular graphics: *ORTEP-3* (Farrugia, 1997 ▶); software used to prepare material for publication: *publCIF* (Westrip, 2010 ▶).

## Supplementary Material

Crystal structure: contains datablocks I, global, New_Global_Publ_Block. DOI: 10.1107/S1600536810038845/fj2341sup1.cif

Structure factors: contains datablocks I. DOI: 10.1107/S1600536810038845/fj2341Isup2.hkl

Additional supplementary materials:  crystallographic information; 3D view; checkCIF report

## Figures and Tables

**Fig. 1.**
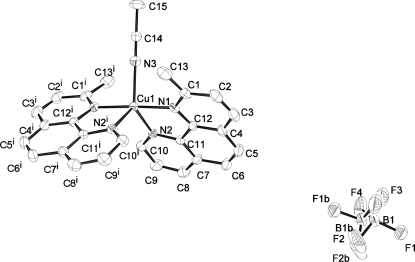
ORTEP plot indicating atom labeling scheme. Labels with an "a" suffix indicate symmetry equivalents of the corresponding atom numbers. Displacement ellipsoids are shown at the 50% probability level and H atoms have been omitted for clarity. The model for anion disorder is indicated and "b" suffixes correspond to lower-occupancy (19%) positions.

**Fig. 2.**
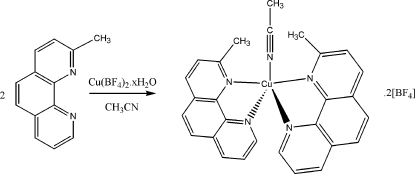
The preparation of the title compound and the structure of the cation.

## supplementary crystallographic information

### Comment

The structure represents the first example of a copper coordination complex containing the 2-methyl phenanthroline ligand, and is part of a continuing study of the structural influence of substitutuents at the proximal positions of the phenanthroline ligand. The structure exhibits 2-fold rotational symmetry at the copper center, with the copper atom and ligated acetonitrile ligand being located on a twofold axis of the unit cell. The methyl groups on the phenanthroline ligand are located close to the N atom of the acetonitrile ligand, and steric interactions between the atoms are most likely responsible for the observed increase in reduction potential for the 2-methyl phenanthroline complex relative to the unsubstituted analog (James, 1961). Nevertheless, the complex does not exhibit the substantial distortion of the copper coordination sphere observed in the analogous 2,9-dimethyl complex (ref, Watton 2009). The BF_4_^-^ counterions exhibit a two-site disorder, refinement of which indicated an approximately 81:19% occupancy of the two sites.

### Experimental

Crystals were grown by vapor diffusion of ether into an acetonitrile solution prepared by addition of 0.041 g (0.20 mmol, ~ 2.1 equivalents) of ligand to 0.024 g (*ca* 0.1 mmol, ~ 1 equivalent) of Cu(BF_4_)_2_.xH_2_O. Yield ~40%. (It should be noted that since the composition of Cu(BF_4_)_2_.xH_2_O is not well defined, the relative amounts of Cu^-^ and ligand, as well as the overall yield of the reaction are correspondingly uncertain).

### Crystal data

**Table:**

[Cu(C_2_H_3_N)(C_13_H_10_N_2_)_2_](BF_4_)_2_	*F*(000) = 1348
*M**_r_* = 666.67	*D*_x_ = 1.597 Mg m^−^^3^
Monoclinic, *C*2/*c*	Melting point: 573 K
Hall symbol: -C2yc	Mo *K*α radiation, λ = 0.71073 Å
*a* = 25.0665 (11) Å	Cell parameters from 18534 reflections
*b* = 8.8120 (1) Å	θ = 3.8–34.6°
*c* = 16.8419 (14) Å	µ = 0.87 mm^−^^1^
β = 131.824 (8)°	*T* = 293 K
*V* = 2772.2 (3) Å^3^	Block, green
*Z* = 4	0.25 × 0.2 × 0.15 mm

### Data collection

**Table:**

Oxford Diffraction Sapphire 3 CCD diffractometer	5578 independent reflections
Radiation source: fine-focus sealed tube	4442 reflections with *I* > 2σ(*I*)
graphite	*R*_int_ = 0.016
ω scans	θ_max_ = 34.7°, θ_min_ = 4.0°
Absorption correction: multi-scan (*CrysAlis RED*; Oxford Diffraction, 2006)	*h* = −39→40
*T*_min_ = 0.784, *T*_max_ = 1	*k* = −14→13
30296 measured reflections	*l* = −26→26

### Refinement

**Table:**

Refinement on *F*^2^	Primary atom site location: structure-invariant direct methods
Least-squares matrix: full	Secondary atom site location: difference Fourier map
*R*[*F*^2^ > 2σ(*F*^2^)] = 0.040	Hydrogen site location: inferred from neighbouring sites
*wR*(*F*^2^) = 0.120	H-atom parameters constrained
*S* = 1.08	*w* = 1/[σ^2^(*F*_o_^2^) + (0.0639*P*)^2^ + 2.765*P*] where *P* = (*F*_o_^2^ + 2*F*_c_^2^)/3
5578 reflections	(Δ/σ)_max_ < 0.001
219 parameters	Δρ_max_ = 1.07 e Å^−^^3^
6 restraints	Δρ_min_ = −0.81 e Å^−^^3^

### Special details

**Table:**

Geometry. All e.s.d.'s (except the e.s.d. in the dihedral angle between two l.s. planes) are estimated using the full covariance matrix. The cell e.s.d.'s are taken into account individually in the estimation of e.s.d.'s in distances, angles and torsion angles; correlations between e.s.d.'s in cell parameters are only used when they are defined by crystal symmetry. An approximate (isotropic) treatment of cell e.s.d.'s is used for estimating e.s.d.'s involving l.s. planes.
Refinement. Refinement of *F*^2^ against ALL reflections. The weighted *R*-factor *wR* and goodness of fit *S* are based on *F*^2^, conventional *R*-factors *R* are based on *F*, with *F* set to zero for negative *F*^2^. The threshold expression of *F*^2^ > σ(*F*^2^) is used only for calculating *R*-factors(gt) *etc*. and is not relevant to the choice of reflections for refinement. *R*-factors based on *F*^2^ are statistically about twice as large as those based on *F*, and *R*- factors based on ALL data will be even larger.

### Fractional atomic coordinates and isotropic or equivalent isotropic displacement parameters (Å^2^)

**Table:**

	*x*	*y*	*z*	*U*_iso_*/*U*_eq_	Occ. (<1)
C1	0.13529 (8)	1.05662 (18)	0.79172 (12)	0.0273 (3)	
C2	0.17740 (9)	1.0448 (2)	0.76412 (15)	0.0338 (3)	
H2	0.2207	1.0967	0.8041	0.041*	
C3	0.15571 (9)	0.9591 (2)	0.68020 (15)	0.0338 (3)	
H3	0.1844	0.9503	0.6638	0.041*	
C4	0.08932 (8)	0.88348 (17)	0.61807 (13)	0.0268 (3)	
C5	0.06222 (10)	0.7871 (2)	0.52973 (15)	0.0335 (3)	
H5	0.0894	0.7721	0.5111	0.040*	
C6	−0.00201 (10)	0.7173 (2)	0.47264 (14)	0.0325 (3)	
H6	−0.0181	0.6543	0.4162	0.039*	
C7	−0.04559 (8)	0.73991 (17)	0.49864 (12)	0.0257 (3)	
C8	−0.11375 (9)	0.6737 (2)	0.44131 (13)	0.0332 (3)	
H8	−0.1322	0.6085	0.3847	0.040*	
C9	−0.15234 (9)	0.7061 (2)	0.46972 (14)	0.0340 (3)	
H9	−0.1975	0.6637	0.4322	0.041*	
C10	−0.12360 (8)	0.80361 (17)	0.55573 (12)	0.0267 (3)	
H10	−0.1506	0.8253	0.5740	0.032*	
C11	−0.02038 (7)	0.83494 (15)	0.58437 (10)	0.0198 (2)	
C12	0.04852 (7)	0.90451 (15)	0.64633 (11)	0.0205 (2)	
C13	0.16252 (10)	1.1435 (2)	0.88823 (14)	0.0377 (4)	
H13A	0.1605	1.2502	0.8748	0.057*	
H13B	0.2112	1.1148	0.9471	0.057*	
H13C	0.1335	1.1217	0.9050	0.057*	
C14	0.0000	1.3587 (2)	0.7500	0.0282 (4)	
C15	0.0000	1.5232 (3)	0.7500	0.0525 (9)	
H15A	−0.0443	1.5595	0.7279	0.079*	0.50
H15B	0.0051	1.5595	0.7016	0.079*	0.50
H15C	0.0392	1.5595	0.8205	0.079*	0.50
Cu1	0.0000	0.99458 (2)	0.7500	0.01924 (7)	
N1	0.07141 (6)	0.98891 (12)	0.73210 (10)	0.0208 (2)	
N2	−0.05911 (6)	0.86627 (13)	0.61224 (9)	0.0207 (2)	
N3	0.0000	1.2302 (2)	0.7500	0.0303 (4)	
B1	0.18636 (13)	0.4493 (3)	0.30558 (19)	0.0314 (4)	0.810 (3)
F1	0.19869 (8)	0.38867 (18)	0.24324 (13)	0.0455 (4)	0.810 (3)
F2	0.1938 (6)	0.3381 (6)	0.3661 (9)	0.1162 (15)	0.810 (3)
F3	0.2324 (2)	0.5670 (4)	0.3570 (3)	0.0722 (10)	0.810 (3)
F4	0.1172 (3)	0.5089 (5)	0.2417 (3)	0.0696 (13)	0.810 (3)
B1B	0.1736 (6)	0.4482 (13)	0.3248 (8)	0.0314 (4)	0.190 (3)
F1B	0.1694 (4)	0.4945 (7)	0.4000 (6)	0.0455 (4)	0.190 (3)
F2B	0.193 (3)	0.306 (3)	0.363 (4)	0.1162 (15)	0.190 (3)
F3B	0.2359 (10)	0.532 (2)	0.3811 (16)	0.0722 (10)	0.190 (3)
F4B	0.1126 (13)	0.463 (3)	0.2259 (18)	0.0696 (13)	0.190 (3)

### Atomic displacement parameters (Å^2^)

**Table:**

	*U*^11^	*U*^22^	*U*^33^	*U*^12^	*U*^13^	*U*^23^
C1	0.0216 (6)	0.0254 (6)	0.0285 (7)	−0.0038 (5)	0.0140 (5)	0.0041 (5)
C2	0.0228 (6)	0.0349 (8)	0.0405 (9)	−0.0028 (6)	0.0198 (6)	0.0081 (7)
C3	0.0280 (7)	0.0370 (8)	0.0432 (9)	0.0026 (6)	0.0265 (7)	0.0097 (7)
C4	0.0284 (6)	0.0272 (6)	0.0321 (7)	0.0056 (5)	0.0233 (6)	0.0083 (5)
C5	0.0408 (8)	0.0371 (8)	0.0385 (8)	0.0077 (7)	0.0331 (8)	0.0055 (7)
C6	0.0425 (9)	0.0337 (8)	0.0309 (7)	0.0045 (6)	0.0284 (7)	−0.0005 (6)
C7	0.0298 (6)	0.0244 (6)	0.0234 (6)	0.0008 (5)	0.0179 (6)	−0.0015 (5)
C8	0.0348 (8)	0.0312 (7)	0.0282 (7)	−0.0057 (6)	0.0188 (6)	−0.0093 (6)
C9	0.0271 (7)	0.0333 (8)	0.0340 (8)	−0.0096 (6)	0.0173 (6)	−0.0112 (6)
C10	0.0222 (6)	0.0266 (6)	0.0303 (7)	−0.0040 (5)	0.0171 (6)	−0.0049 (5)
C11	0.0214 (5)	0.0176 (5)	0.0208 (5)	0.0013 (4)	0.0143 (5)	0.0014 (4)
C12	0.0208 (5)	0.0194 (5)	0.0230 (6)	0.0018 (4)	0.0153 (5)	0.0041 (4)
C13	0.0314 (8)	0.0371 (8)	0.0319 (8)	−0.0150 (7)	0.0158 (7)	−0.0063 (6)
C14	0.0373 (11)	0.0202 (8)	0.0382 (11)	0.000	0.0298 (10)	0.000
C15	0.068 (2)	0.0182 (10)	0.100 (3)	0.000	0.068 (2)	0.000
Cu1	0.01965 (11)	0.01729 (11)	0.02048 (11)	0.000	0.01326 (9)	0.000
N1	0.0189 (5)	0.0198 (5)	0.0218 (5)	−0.0012 (4)	0.0128 (4)	0.0020 (4)
N2	0.0198 (5)	0.0192 (5)	0.0235 (5)	−0.0016 (4)	0.0146 (4)	−0.0016 (4)
N3	0.0415 (10)	0.0213 (8)	0.0366 (10)	0.000	0.0296 (9)	0.000
B1	0.0278 (10)	0.0394 (11)	0.0316 (10)	0.0043 (8)	0.0217 (8)	0.0098 (9)
F1	0.0439 (7)	0.0483 (8)	0.0544 (9)	−0.0133 (6)	0.0370 (7)	−0.0170 (6)
F2	0.1160 (18)	0.129 (3)	0.153 (2)	0.061 (3)	0.1105 (19)	0.109 (3)
F3	0.0757 (12)	0.081 (2)	0.098 (2)	−0.0216 (14)	0.0735 (16)	−0.0299 (17)
F4	0.0408 (10)	0.124 (4)	0.0501 (17)	0.040 (2)	0.0330 (13)	0.0346 (19)
B1B	0.0278 (10)	0.0394 (11)	0.0316 (10)	0.0043 (8)	0.0217 (8)	0.0098 (9)
F1B	0.0439 (7)	0.0483 (8)	0.0544 (9)	−0.0133 (6)	0.0370 (7)	−0.0170 (6)
F2B	0.1160 (18)	0.129 (3)	0.153 (2)	0.061 (3)	0.1105 (19)	0.109 (3)
F3B	0.0757 (12)	0.081 (2)	0.098 (2)	−0.0216 (14)	0.0735 (16)	−0.0299 (17)
F4B	0.0408 (10)	0.124 (4)	0.0501 (17)	0.040 (2)	0.0330 (13)	0.0346 (19)

### Geometric parameters (Å, °)

**Table:**

C1—N1	1.3356 (18)	C12—N1	1.3652 (18)
C1—C2	1.413 (2)	C13—H13A	0.9600
C1—C13	1.488 (2)	C13—H13B	0.9600
C2—C3	1.356 (3)	C13—H13C	0.9600
C2—H2	0.9300	C14—N3	1.133 (3)
C3—C4	1.409 (2)	C14—C15	1.449 (3)
C3—H3	0.9300	C15—H15A	0.9600
C4—C12	1.3985 (19)	C15—H15B	0.9600
C4—C5	1.431 (2)	C15—H15C	0.9600
C5—C6	1.353 (3)	Cu1—N1^i^	2.0042 (12)
C5—H5	0.9300	Cu1—N1	2.0043 (12)
C6—C7	1.436 (2)	Cu1—N2	2.0669 (12)
C6—H6	0.9300	Cu1—N2^i^	2.0669 (12)
C7—C11	1.4030 (19)	Cu1—N3	2.0759 (19)
C7—C8	1.411 (2)	B1—F2	1.334 (6)
C8—C9	1.365 (3)	B1—F3	1.351 (4)
C8—H8	0.9300	B1—F1	1.384 (3)
C9—C10	1.401 (2)	B1—F4	1.397 (5)
C9—H9	0.9300	B1B—F4B	1.32 (3)
C10—N2	1.3312 (18)	B1B—F2B	1.344 (16)
C10—H10	0.9300	B1B—F3B	1.381 (15)
C11—N2	1.3589 (17)	B1B—F1B	1.399 (11)
C11—C12	1.4297 (18)		
			
N1—C1—C2	120.27 (15)	C1—C13—H13C	109.5
N1—C1—C13	119.85 (14)	H13A—C13—H13C	109.5
C2—C1—C13	119.87 (14)	H13B—C13—H13C	109.5
C3—C2—C1	121.05 (15)	N3—C14—C15	180.000 (3)
C3—C2—H2	119.5	C14—C15—H15A	109.5
C1—C2—H2	119.5	C14—C15—H15B	109.5
C2—C3—C4	119.43 (15)	H15A—C15—H15B	109.5
C2—C3—H3	120.3	C14—C15—H15C	109.5
C4—C3—H3	120.3	H15A—C15—H15C	109.5
C12—C4—C3	117.02 (15)	H15B—C15—H15C	109.5
C12—C4—C5	118.98 (14)	N1^i^—Cu1—N1	177.15 (6)
C3—C4—C5	124.00 (14)	N1^i^—Cu1—N2	96.41 (5)
C6—C5—C4	121.56 (14)	N1—Cu1—N2	82.02 (5)
C6—C5—H5	119.2	N1^i^—Cu1—N2^i^	82.02 (5)
C4—C5—H5	119.2	N1—Cu1—N2^i^	96.41 (5)
C5—C6—C7	120.47 (15)	N2—Cu1—N2^i^	113.67 (7)
C5—C6—H6	119.8	N1^i^—Cu1—N3	91.43 (3)
C7—C6—H6	119.8	N1—Cu1—N3	91.43 (3)
C11—C7—C8	117.39 (13)	N2—Cu1—N3	123.16 (3)
C11—C7—C6	118.96 (14)	N2^i^—Cu1—N3	123.16 (3)
C8—C7—C6	123.65 (14)	C1—N1—C12	119.08 (13)
C9—C8—C7	119.42 (14)	C1—N1—Cu1	128.24 (11)
C9—C8—H8	120.3	C12—N1—Cu1	112.67 (9)
C7—C8—H8	120.3	C10—N2—C11	118.39 (12)
C8—C9—C10	119.64 (15)	C10—N2—Cu1	130.48 (10)
C8—C9—H9	120.2	C11—N2—Cu1	110.88 (9)
C10—C9—H9	120.2	C14—N3—Cu1	180.000 (1)
N2—C10—C9	122.38 (14)	F2—B1—F3	116.7 (6)
N2—C10—H10	118.8	F2—B1—F1	107.9 (5)
C9—C10—H10	118.8	F3—B1—F1	104.8 (2)
N2—C11—C7	122.78 (13)	F2—B1—F4	109.5 (5)
N2—C11—C12	117.02 (12)	F3—B1—F4	107.2 (3)
C7—C11—C12	120.20 (12)	F1—B1—F4	110.5 (2)
N1—C12—C4	123.06 (13)	F4B—B1B—F2B	116 (3)
N1—C12—C11	117.17 (11)	F4B—B1B—F3B	130.2 (18)
C4—C12—C11	119.77 (13)	F2B—B1B—F3B	107 (2)
C1—C13—H13A	109.5	F4B—B1B—F1B	113.1 (12)
C1—C13—H13B	109.5	F2B—B1B—F1B	94 (2)
H13A—C13—H13B	109.5	F3B—B1B—F1B	87.4 (9)

Symmetry codes: (i) −*x*, *y*, −*z*+3/2.
